# Computer-aided drug discovery: historical foundations, practical AI tools, and emerging ethical considerations

**DOI:** 10.3389/fphar.2026.1866562

**Published:** 2026-06-24

**Authors:** Rebekah Clarke, Giuseppe Palazzo, Jiri Ruzicka, Salvatore Ferla, Marcella Bassetto

**Affiliations:** 1 Medical School, Faculty of Medicine, Health and Life Science, Swansea University, Swansea, United Kingdom; 2 School of Pharmacy and Pharmaceutical Sciences, Cardiff University, Cardiff, United Kingdom

**Keywords:** ADMET prediction, artificial intelligence, computational pharmacology, computer-aided drug discovery, machine learning, virtual screening

## Abstract

Computer-aided drug discovery (CADD) has become an integral component of modern drug development, supporting hit identification, lead optimisation, and candidate refinement across both academia and industry. Over 4 decades of methodological progress have contributed to the discovery or optimisation of several approved therapeutics, establishing CADD as a crucial element of drug research. In parallel, recent advances in artificial intelligence (AI) and machine learning (ML) have introduced a new generation of practical tools that offer improved predictive performance, accessible software implementations, and increasing integration into everyday drug discovery and pharmacology workflows. This review provides a concise historical overview of CADD, with an updated account of approved drugs and clinical-stage candidates whose discovery or optimisation has involved computational methods and highlights a curated set of contemporary AI and ML tools that are readily usable by non-specialists. In addition, we compile and analyse two comprehensive, practice-oriented resources: a collection of cloud-based virtual-screening platforms, and an extensive suite of freely accessible ADMET prediction tools, offering medicinal chemists a consolidated guide to open-source computational workflows. Finally, we discuss emerging ethical considerations, including data bias, transparency, computational costs, and the environmental impact of large-scale models, outlining responsible paths for the continued adoption of AI in drug discovery.

## 1 Introduction

The use of computer-aided drug discovery (CADD) has expanded dramatically over the past 40 years, with early applications emerging in the 1980s. (Gund et al., 1980). Since then, CADD has grown into a powerful and interdisciplinary component of modern drug discovery, contributing to the identification and optimisation of hit compounds across diverse therapeutic areas. Historically, the journey toward novel therapeutics was often lengthy, resource-intensive, and largely dependent on serendipity or iterative trial-and-error experimentation. In contrast, CADD has transformed this landscape by enabling high-throughput, precise evaluation of large chemical libraries, and facilitating exploration of vast chemical space within greatly reduced timeframes.

**FIGURE 1:**
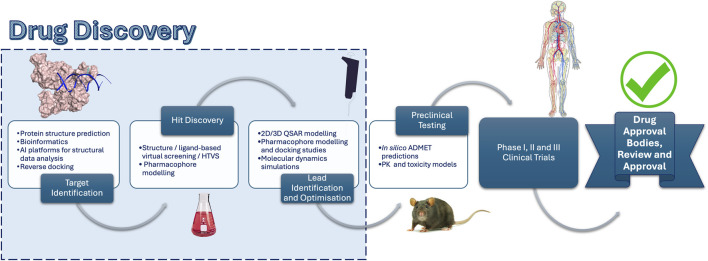
The applications of CADD in the drug discovery pipeline.^1^

The foundations of CADD lie in the integration of complex biological systems with the predictive capabilities of computational algorithms, supported by the development of large curated chemical and biological databases (Drews, 2000). Over recent decades, CADD has expanded to influence nearly every stage of the drug discovery pipeline, from initial target identification and validation to lead optimisation and preclinical assessment (Figure 1) (Wang et al., 2024) Its ability to predict which molecular candidates are most likely to display favourable therapeutic profiles allows researchers to prioritise high-potential compounds early in the process, thereby increasing efficiency and reducing time and resource requirements (Nascimento et al., 2022). This predictive capacity is especially advantageous when rapid drug development is needed, such as during emerging infectious disease outbreaks.

During the COVID-19 pandemic, CADD approaches played a significant role in accelerating early identification of potential antiviral agents, primarily through virtual screening of small-molecule libraries and the repurposing of existing FDA-approved drugs (Yang et al., 2024). Techniques including molecular docking, molecular dynamics simulations, and AI-based modelling were applied to identify compounds targeting key viral proteins, such as the spike protein and main protease, and to support the design of stabilised vaccine constructs (Yang et al., 2024). Although these efforts did not yield novel approved drugs for COVID-19 to date, they demonstrated the potential of computational methods to guide rapid therapeutic development, and highlighted the importance of continuously improving computational tools, expanding datasets, and adopting innovative methodologies for future global health challenges (Yang et al., 2024; Tamanna et al., 2021).

CADD has evolved considerably in parallel with advances in computational power, algorithm development, and molecular biology (Supplementary Figure S1) (Tamanna et al., 2021). *In silico* strategies offer compelling advantages by reducing reliance on animal models for early testing, decreasing laboratory costs, and accelerating the overall research timeline (An et al., 2025). In its early stages, CADD relied primarily on quantitative structure-activity relationships (QSAR), which correlated chemical structure with biological activity and provided the groundwork for predictive modelling (Cherkasov et al., 2014; Bassani and Moro, 2023). Subsequent advances in high-performance computing and molecular simulation enabled more sophisticated methodologies, including structure-based drug design (SBDD) and ligand-based drug design (LBDD) methods. SBDD leverages detailed structural information about biological targets, made possible through techniques such as X-ray crystallography and nuclear magnetic resonance (NMR) spectroscopy, to design compounds tailored to specific binding sites (van Montfort and Workman, 2017). LBDD, in contrast, uses known bioactive molecules to infer pharmacophoric or structural features necessary for activity when target structures are unavailable (Chayan et al., 2011).

More recently, CADD has entered a new phase through the integration of artificial intelligence (AI) and machine learning (ML) (Kim et al., 2020). These techniques enhance data analysis, enable more accurate and efficient prediction of molecular properties, and provide new avenues for compound generation and optimisation. AI and ML models are capable of extracting patterns from large collections of molecular, biological, and pharmacological data, offering insights that were previously inaccessible and marking the beginning of a new era of data-driven drug discovery.

In this review, we provide a concise historical overview of the development of CADD, focused on an updated and curated account of approved drugs and clinical-stage candidates whose discovery or optimisation involved computational methods. We then highlight a selection of recent and practically accessible AI and machine-learning tools that are increasingly integrated into modern medicinal chemistry workflows, providing two consolidated, practice-ready resources for researchers: a curated collection of cloud-based platforms for structure-based virtual screening, and a comprehensive set of freely available ADMET-prediction tools, enabling immediate implementation of CADD and AI methods in drug-discovery projects.

Finally, we discuss emerging ethical and environmental considerations associated with AI-driven drug discovery.

## 2 Therapeutics enabled by CADD approaches

CADD has become increasingly important in precision medicine (Santus et al., 2021), supporting the development of therapies tailored to individual genetic profiles and disease mechanisms. By helping to prioritise compounds for synthesis and experimental testing, CADD lessens the environmental and ethical burden of early-stage discovery (Bassani and Moro, 2023), while supporting safety and regulatory compliance by enabling early prediction of potential toxicity or efficacy liabilities during development. In addition to this, the adoption of computational methods reduces issues accompanying traditional workflows, such as unnecessary reagent consumption associated with chemical synthesis, and reduces the need for *in vivo* experimentation.

Several approved drugs, along with a growing number of clinical candidates, owe aspects of their discovery or optimisation to CADD methodologies. Table 1 presents a representative subset of landmark therapeutics for which computational methods played a documented role, while the full dataset is provided in Supplementary Table S1. The extent of computational influence varies widely: in some programmes, such as HIV protease inhibitors and early kinase inhibitors, SBDD provided decisive structural guidance, whereas in others CADD primarily supported medicinal chemistry through SAR rationalisation, conformational analysis, or exploratory modelling. Table 1 is therefore interpretive rather than exhaustive, illustrating how computation complemented broader experimental efforts, rather than implying that CADD alone enabled each success.

**TABLE 1:** Representative landmark CADD-enabled approved drugs.

Drug	Therapeutic application	CADD approach	FDA approval date
Captopril	Angiotensin-converting enzyme (ACE) inhibitor (hypertension)	QSAR	1981
Losartan	Angiotensin II receptor antagonist (hypertension)	LBDD, pharmacophore modelling and SAR optimisations	1995
Saquinavir	Inhibitor of HIV I protease (HIV)	SBDD	1995
Indinavir	Inhibitor of HIV I protease (HIV)	SBDD with molecular dynamics and X-ray crystallography	1996
Ritonavir	Inhibitor of HIV I protease (HIV)	SBDD, LBDD, SAR optimisations	1996
Zanamivir	Antiviral (influenza A and B)	SBDD	1999
Imatinib	Tyrosine kinase inhibitor (cancers)	SBDD	2001
Gefitinib	EGFR tyrosine kinase inhibitor (certain breast, lung and other cancers)	Structure-based VS	2003
Raltegravir	HIV integrase inhibitor (HIV)	SBDD with MD simulations	2007
Venetoclax	BCL-2 inhibitor (leukaemia treatment)	FBDD	2016
Erdafitinib	FGFR inhibitor (metastatic or locally advanced bladder cancer with an FGFR3 or FGFR2 alteration)	FBDD and SBDD	2019
Sotorasib	RAS GTPase inhibitor for (non-small cell lung cancer)	FBDD	2021

MD–Molecular Dynamics; LBDD–Ligand-Based Drug Design; PBDD–Pharmacophore-Based Drug Design; SBDD–Structure-Based Drug Design; VS–Virtual Screening; SAR–Structure Activity Relationship; QSAR–Quantitative Structural Activity Relationship.

Examination of Table 1 and Supplementary Table S1 reveals recurring methodological patterns. SBDD is the most frequently represented approach, reflecting both the maturation of crystallography during the 1990s and the value of structural insight in antiviral and kinase programmes. Later approvals, including imatinib, gefitinib, raltegravir, and talazoparib, further demonstrate how structural information enabled targeted optimisation, though typically alongside extensive medicinal chemistry. LBDD methods also appear prominently, particularly in cases where structural data were initially limited and computational modelling aided scaffold refinement. PBDD appears infrequently, while QSAR, foundational in the early computational era, now tends to function within hybrid pipelines rather than as a standalone driver. FBDD features strongly in modern campaigns, as seen for venetoclax, erdafitinib, and sotorasib, highlighting its increasing role in fragment-based optimisation.

Taken together, Table 1 and Supplementary Table S1 show that successful discovery efforts have relied on context-dependent combinations of computational and experimental approaches, with strategies shifting as structural information evolved. For medicinal chemists, the key lesson is that CADD contributes most effectively when integrated iteratively with empirical SAR and mechanistic insight.

Despite the clear value of CADD in supporting successful discoveries, the wider drug-development landscape remains constrained by high attrition rates and substantial resource requirements. Drug discovery has traditionally been a lengthy and resource-intensive endeavour, with a low reward-to-risk ratio (Berdigaliyev and Aljofan, 2020). Attrition, the failure of drug candidates at various stages of development, remains a major challenge, with approximately nine out of ten molecules failing during Phase I, II, or III clinical trials, or at the regulatory approval stage (Sun et al., 2022). Analyses of clinical trial outcomes between 2010 and 2017 indicate that the primary reasons for attrition include lack of clinical efficacy (40%–50%), unmanageable toxicity (30%), poor drug-like properties (10%–15%), and strategic or commercial considerations (10%) (Helen Dowden and Munro, 2019; Harrison, 2016). A further contributor is the translational gap between preclinical models and human physiology, which can result in unexpected failures during clinical evaluation (Martić-Kehl et al., 2012).

These high failure rates substantially increase the average cost of bringing a single drug to market, as resources must be allocated to compensate for multiple unsuccessful candidates (Moreno and Pearson, 2013). Late-stage failures are particularly costly, often representing years of investment and hundreds of millions of dollars. Beyond financial considerations, such inefficiencies raise ethical questions regarding the responsible use of research funding and experimental resources. As a result, the traditional drug discovery pipeline is increasingly viewed as unsustainable due to high attrition, rising costs, and prolonged timelines.

By contrast, data-driven approaches, including those enabled by CADD, offer opportunities to mitigate these challenges. Computational modelling supports the early identification of promising candidates, the prediction of drug-like properties, and more informed decision-making throughout the pipeline. These advances may enable more informed early-stage decision-making, but there is currently limited evidence that they reduce clinical attrition or accelerate downstream development; such impacts remain future expectations rather than established outcomes.

## 3 Artificial intelligence and machine learning in drug design

**FIGURE 2:**
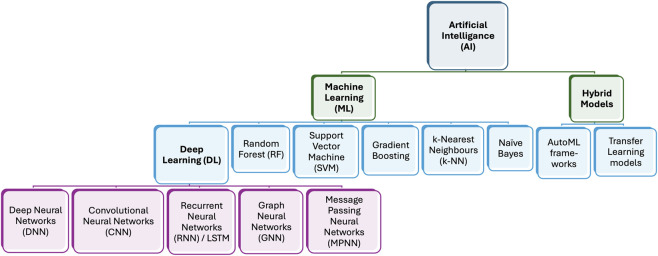
Architectural depiction of ML/DL algorithms and hybrid models within AI.

Over the past decade, AI and ML have become progressively incorporated into modern CADD. These approaches build on traditional ligand- and structure-based methods by analysing complex biological and chemical datasets, and generating predictive models that support key early-stage design tasks (Zhang et al., 2025). AI techniques now contribute to target prioritisation, structure prediction, virtual screening, *de novo* molecular design, ADMET profiling, and synthesis planning across both academia and industry. ML algorithms learn predictive patterns directly from data, while deep learning (DL) extends this capability through multi-layer neural networks that can operate on raw molecular graphs, sequences, or images (Figure 2). The following sections summarise major applications of AI and ML in drug design, drawing on representative studies and practical tools, and outlining the opportunities and limitations of these rapidly evolving methodologies. Recent advances in generative AI and foundation models represent an important development within AI-driven drug discovery (Pang et al., 2024). Generative architectures, including variational autoencoders (VAEs), graph-based models, reinforcement learning (RL) approaches, transformers and diffusion models, can propose novel molecular structures while optimising multiple parameters such as predicted potency, selectivity, physicochemical properties, ADMET behaviour and synthetic feasibility (Gangwal et al., 2024). Foundation models trained on large molecular, protein or multimodal biological datasets further enable transfer learning across related drug-discovery tasks, reducing the need for extensive target-specific training data (Huang et al., 2024a; Xu et al., 2025). These approaches are now being applied to *de novo* molecular generation, protein and complex structure prediction, synthesis planning, target prioritisation and multiparameter lead optimisation (Xu et al., 2025). However, despite their promise, generative and foundation models remain hypothesis-generating tools that require rigorous experimental validation, careful uncertainty assessment and medicinal chemistry interpretation.

### 3.1 Target identification

Target identification is the foundational stage of drug discovery, involving the recognition of biological macromolecules or pathways associated with disease. Traditional target-identification approaches, such as experimental biology, genetic studies, and network-based analyses, have been highly successful but are often labour-intensive, time-consuming, and dependent on the generation of large experimental datasets (Schenone et al., 2013). AI-based approaches have emerged as complementary tools that can integrate diverse biological data sources, including genomics, transcriptomics, proteomics, and clinical information, to prioritise targets that may have therapeutic relevance (Pun et al., 2023). Rather than replacing experimental methods, these approaches help researchers focus resources on the most promising biological hypotheses for further investigation.

A notable advancement in this area is the Functional Representation of Gene Signatures (FRoGS) framework, which enhances target prediction by analysing gene expression signatures using DL-based functional embeddings (Chen et al., 2024). Trained on large transcriptomic datasets such as those from the L1000 platform, FRoGS uses deep-learning-derived representations of transcriptomic data to improve prediction of drug-target relationships, and has demonstrated improved performance compared with conventional signature-matching approaches (Chen et al., 2024).

Using large-scale clinical and genomic datasets, Cosentino *et al.* utilised a deep convolutional network trained on raw spirogram data to identify 67 previously unrecognised COPD-associated loci (Cosentino et al., 2023). This illustrates how AI can reveal disease mechanisms and generate novel target hypotheses for therapeutic development.

AI tools have also accelerated efforts to identify small molecules targeting microRNAs (miRNAs), historically limited by the need for bespoke high-throughput assays. (Gabr and Brogi, 2020). Early computational tools such as Inforna matched RNA motifs to small-molecule inhibitors using curated RNA structure libraries, (Disney et al., 2016), while recent ML approaches, including the random forest-based RFSMMA (Wang et al., 2019), and the DL-based ZeSTa (Gao et al., 2024), predict miRNA-small molecule interactions and binding nucleotides. Random forests are ensembles of decision trees that reduce overfitting by averaging predictions across many diverse trees, while DL models can learn sequence-structure relationships directly from data without hand-crafted features. Although experimental validation remains limited, these models demonstrate growing potential for accelerating miRNA-targeted drug discovery.

Despite these successes, AI-based target identification remains highly dependent on the quality and completeness of the underlying biological data (Wenteler et al., 2024). Furthermore, AI models primarily generate hypotheses and prioritise candidate targets; their predictions require extensive experimental validation before causal disease relevance and therapeutic potential can be established (Pun et al., 2026).

### 3.2 Structure prediction

When experimental structural data are unavailable, accurate protein modelling is critical for enabling structure-based drug design (SBDD). Classical predictive methods include *ab initio* modelling, threading, and homology modelling (Ali et al., 2024). The field was transformed by AlphaFold2, whose performance at CASP14 marked a step-change in structural prediction accuracy (Jumper et al., 2021). Trained on large protein sequence and structure datasets using DL, AlphaFold2 produced near-experimental-quality models (median backbone accuracy 0.96 Å r.m.s.d._95_) (Jumper et al., 2021) and has since been widely adopted for virtual screening, docking, and therapeutic design. At a high level, AlphaFold-like systems learn residue-residue geometries (distances/orientations) and iteratively refine 3D coordinates with attention mechanisms that capture long-range sequence dependencies.

AlphaFold3 expanded these capabilities (AlphaFold Server, 2024) by modelling proteins, nucleic acids, and small-molecule ligands, and demonstrated ∼50% improved accuracy over previous methods on benchmarks such as PoseBusters (Abramson et al., 2024). These advances have facilitated novel drug-discovery applications, including the identification of ISM042-2-048, a CDK20 inhibitor for hepatocellular carcinoma discovered using PandaOmics and Chemistry42 in combination with AlphaFold-based structures (Ren et al., 2023).

Beyond AlphaFold, novel predictive models such as trRosettaX-Single use supervised transformer-based protein language models to generate accurate 2D geometries and 3D structures from single sequences with reduced computational cost (Wang et al., 2022). Transformers rely on self-attention, which weighs relationships between all positions in a sequence, enabling the model to capture long-range dependencies that are difficult for recurrent or convolutional architectures. Despite these successes, static structural predictions represent only one conformational state, and predicting physiologically relevant ensembles remains a major challenge (Cui et al., 2025). Hybrid approaches that combine AlphaFold-like predictions with molecular dynamics, generative modelling, or experimental constraints are emerging to address protein flexibility and dynamics (Cui et al., 2025).

For medicinal chemists, AlphaFold-based structures are therefore best treated as hypotheses rather than definitive receptor models (Borkakoti and Thornton, 2023). Although models can provide a valuable basis for docking, binding-site analysis and target prioritisation, they are not experimentally determined ligand-bound structures and may not reliably capture induced fit, cryptic pockets, allosteric states, protonation states, cofactors, metal coordination, post-translational modifications, membrane context or protein flexibility (Holcomb et al., 2023). These limitations are particularly relevant for molecular docking and virtual screening, where small errors in side-chain orientation, pocket hydration or loop conformation can substantially affect predicted ligand poses and rankings (Scardino et al., 2023). Confidence metrics such as pLDDT and PAE can help identify unreliable regions, but they do not directly validate ligandability or binding affinity (Jumper et al., 2021). Before docking or hit identification, they should be refined through practical preparation steps, including addition of missing cofactors or ions, protonation-state assignment, steric-clash correction, binding-site inspection, comparison with homologous ligand-bound structures, and, where appropriate, short molecular-dynamics simulations or ensemble generation.

### 3.3 Machine learning in QSAR

ML, particularly DL, has increasingly shaped QSAR modelling by enabling pattern recognition in large, complex datasets (Tropsha et al., 2024). The first DL-QSAR study was published in 2015, and the field has since expanded to hundreds of applications (Ma et al., 2015). Traditional QSAR relies on predefined descriptors, whereas DL replaces descriptor engineering with learned molecular embeddings generated from SMILES strings, graphs, or molecular images (Tropsha et al., 2024; Ma et al., 2015; Bengio et al., 2013). Graph neural networks (GNNs) are especially useful here, while deep neural network architectures, including multilayer perceptrons, convolutional neural networks (CNNs), and graph convolutional neural networks (GCNNs), have been applied to predict biological activities (Huang et al., 2024b). MIA-QSAR uses CNNs to extract chemical features from molecular images (Khaouane et al., 2023), while federated learning-based QSAR (FL-QSAR) allows institutions to collaboratively train models without sharing raw data (Chen et al., 2020). Federated learning keeps data behind institutional firewalls and aggregates model updates centrally, improving privacy while retaining the benefits of larger effective datasets.

Schrödinger’s DeepAutoQSAR platform automates QSAR model generation using integrated ML and DL frameworks (Deepautoqsar, 2026) offering confidence estimation and atom-wise contributions (Zach Kaplan, 2022). However, the main benchmarking study supporting this platform is a vendor-generated white paper rather than an independent peer-reviewed evaluation, and should therefore be interpreted with caution unless corroborated by external benchmarking studies (Zach Kaplan, 2022; Xu et al., 2024).

Nippa *et al.* demonstrated late-stage lead diversification using geometric DL, training 100 deep QSAR models with the ELECTRA algorithm to predict PI3Kγ inhibition (Nippa et al., 2024). This approach yielded a potent inhibitor (Ki = 63 nM), supporting DL-enabled strategies for automated medicinal chemistry (Mad et al., 2022). Although no approved drugs have been enabled through DL-QSAR to date, advances in accuracy and accessibility suggest that DL-QSAR will contribute increasingly to preclinical pipelines. As a rule of thumb, DL tends to outperform classical ML when datasets are larger, multi-task signals are available, or graph/sequence inputs carry rich structure that hand-crafted descriptors struggle to capture. Similarly, DL-based QSAR becomes most useful when available datasets are moderately large, multi-task endpoints are available, or graph and sequence inputs contain structural information that classical descriptors fail to capture.

### 3.4 Virtual screening

Virtual screening (VS) has become central to hit identification by enabling prioritisation of compounds from large libraries using SBDD or ligand-based approaches (Kimber et al., 2021). DL has been integrated into VS workflows through complex-based and pair-based models that predict binding affinity using protein-ligand structural information or ligand-target pairs (Kimber et al., 2021). In complex-based models, 3D protein-ligand poses are encoded (e.g., as voxel grids) so networks can learn interaction patterns; in pair-based models, ligands are paired with simplified target descriptors to learn cross-target SAR trends. Examples include Interaction Pseudo Fingerprints for DNN training, which improved performance over Glide and AutoDock Vina in SAM MTase inhibitor identification (Li et al., 2019).

3D CNN architectures using voxelised protein-ligand grids (e.g., AtomNet, BindScope, DeepAtom) have outperformed classical scoring functions in several benchmarks, despite high memory demands and grid-boundary limitations (Stepniewska-Dziubinska et al., 2018; Jiménez et al., 2018; Ragoza et al., 2017; Ballester and Mitchell, 2010). Alternative CNN models such as DeepBindRG and DeepVS focus on atomic environments, performing comparably to Vina and Pafnucy (Stepniewska-Dziubinska et al., 2018). SMILES- and sequence-based regression methods such as DeepDTA extend VS to ligand-protein affinity prediction using fixed-length inputs (Öztür et al., 2018).

DL-re-scoring methods provide strong improvements in real VS campaigns. DeepDock contributed to identifying potent NSP14 inhibitors of SARS-CoV-2 (Luo et al., 2025; Gentile et al., 2020), and potent Aβ42 aggregation inhibitors for Alzheimer’s disease, including hits with low‐nM binding affinity after screening 539 million molecules (Brezinova et al., 2026).

RosettaVS, integrating receptor flexibility, outperformed other state-of-the-art SBVS tools and enabled rapid discovery of micromolar ligands for KLHDC2 and NaV1.7, validated by X-ray crystallography (Zhou et al., 2024).

Finally, cloud-based high-throughput VS offers scalable, accessible VS pipelines independent of local hardware, supporting global collaboration and large-scale docking (Table 2). (Olğaç et al., 2019) These platforms facilitate hit discovery, repurposing, and pharmacokinetic profiling through flexible infrastructure.

**TABLE 2:** Currently available cloud-based platforms for virtual screening.

*Platform*	*Description*	*URL*
3decision	Protein structure repository for comprehensive structural data management and advanced analytics (SBDD)	Decision (2026)
Achilles	Blind docking server (workflow)	Achilles Blind Docking Server (2026)
BindScope	Binding prediction tool (SBDD)	PlayMolecule AI Server (2018)
DOCK Blaster	Automated molecular docking-based VS web service (SBDD)	DOCK Blaster Server (2021)
DockingServer	Molecular docking and VS platform (SBDD)	Docking Server (2021)
HDOCK	Protein-protein and protein-DNA/RNA docking based on a hybrid algorithm of template-based modelling and *ab initio* free docking (SBDD)	HDock (2025)
HADDOCK Web Server	Information-driven flexible docking server for the modelling of biomolecular complexes (SBDD)	Haddock (2026)
LigandScout Remote	Interface that integrates advanced pharmacophore and VS with molecular docking tools	Ligand Scout (2026)
mCule	Structure-based VS tools with purchasable chemical libraries (workflow)	mCule (2026)
MTiOpenScreen	Combination of AutoDock and MTiOpenScreen in bioinformatics Mobyle environment (workflow)	MTiOpenScreen (2021)
ParDOCK	All-atom energy-based Monte Carlo, rigid protein ligand docking, implemented in a fully automated, parallel processing mode for binding mode prediction (SBDD)	ParDOCK (2015)
PatchDock	Molecular docking algorithm based on shape complementarity principles (SBDD)	PatchDock (2005)
Polypharmocology Browser 2 (PPB2)	Web server for target prediction for the ligands (LBDD)	Polypharmocology Browser (2018)
ProBiS	Web-based analysis tool for binding site identification (SB/LBDD)	ProBiS (2016)
SwissDock	Web service predicts target protein and small molecule interactions with Attracting Cavities 2.0 (AC) and AutoDock Vina (Vina) (workflow)	Swiss Dock (2024)
ZincPharmer	Pharmacophore search software for screening the purchasable subset of the ZINC database (LBDD)	Zinc Pharmer (2026)

The platforms listed in Table 2 highlight the range of cloud-accessible tools available for virtual screening, from general-purpose docking servers (e.g., SwissDock, DOCK Blaster, DockingServer) to information-driven engines for macromolecular assemblies (HADDOCK, HDOCK, PatchDock) and end-to-end workflows such as MTiOpenScreen, Achilles, and mCule. Ligand-focused utilities (e.g., LigandScout Remote, ZincPharmer, PPB2) further support pharmacophore search and target prediction, while resources such as ProBiS and 3decision aid binding-site identification and structural data organisation. In practice, structure-based servers are most effective when high-quality target structures are available, whereas information-driven tools help address protein-protein or nucleic-acid interfaces, and ligand-based approaches remain valuable where structural data are limited. Workflow servers standardise preparation steps and lower the barrier for non-specialists, though they may limit customisation and require attention to data-governance policies. As with all virtual screening, success depends on careful input preparation, orthogonal validation, and critical inspection of poses, with refinement reserved for the most promising candidates. Overall, Table 2 illustrates how cloud-based screening can combine ligand-based filtering, structure-based docking, and targeted experimental follow-up in a streamlined and accessible manner.

### 3.5 *De novo* drug design

*De novo* design aims to generate novel molecules with specific biological and physicochemical properties (Meyers et al., 2021). Traditional methods are limited by chemical-space size and iterative synthesis requirements. This area has recently been accelerated by foundation-model and diffusion-based approaches, which extend earlier VAE, GAN and reinforcement-learning strategies by learning richer molecular representations from larger chemical and biological datasets (Alakhdar et al., 2024). VAEs and related models map molecules to a continuous latent space and then sample new points that decode to valid structures, while generative adversarial networks (GANs) pit a generator against a discriminator to produce realistic, novel chemotypes (Mouchlis et al., 2021). RL further optimises generated molecules for desired properties. In reinforcement learning, a model receives a reward (e.g., predicted potency, drug-likeness, synthesisability) for each candidate and learns to generate molecules that maximise this reward over many iterations.

The GENTRL model from Insilico Medicine integrates tensorial embeddings with reinforcement learning to rapidly produce drug-like molecules (Zh et al., 2019). This framework discovered several DDR1 inhibitors, including compounds with potent IC_50_ values of 10 and 21 nM, with one lead candidate showing favourable pharmacokinetics in mice (Zh et al., 2019).

Additionally, active learning approaches integrate molecular dynamics (MD) simulations and iterative ML refinement, as demonstrated by Elez *et al.*, who identified BMS-262084 as a potent TMPRSS2 inhibitor (IC_50_ = 1.82 nM) with broad coronavirus activity while reducing screening costs by ∼29-fold (Elez et al., 2025). Active learning closes the loop between modelling and experiments by selecting the next batch of compounds expected to be maximally informative, thereby reducing the number of syntheses and assays required.

### 3.6 ADMET predictions

Accurate prediction of absorption, distribution, metabolism, excretion, and toxicity (ADMET) is crucial for evaluating drug candidates. Traditional ADMET studies are expensive and often performed too late (Ferreira and Andricopulo, 2019). AI enables rapid early-stage ADMET prediction using ML and DL algorithms such as random forests, SVMs, XGB, and DMPNN-based graph networks (Han et al., 2025). Two concepts are key for practice: the applicability domain (the region of chemical space where a model is reliable) and uncertainty estimation (how confident the model is in a given prediction). Reporting both helps avoid over-interpreting out-of-domain results.

Accessible ADMET prediction tools (Table 3) provide user-friendly interfaces for forecasting bioavailability, BBB penetration, metabolic stability, and toxicity (Fu and Chen, 2025).

**TABLE 3:** Open-source AI-facilitated tools for ADMET predictions.

Tool	Model*	Availability
ADME@NCATS	Deep learning (GCNNs) and machine learning algorithms (SVMs, RF)	Free online (ADME, 2024)
ADMET-AI	Chemprop-RDKit graph neural network	Free online (ADMET-AI, 2024)
ADMETlab 3.0	Machine learning (Directed Message Passing Neural Network (DMPNN) framework)	Free online (ADMETlab 3, 2024)
CYProduct	Machine learning approach to predict cytochrome metabolism	Free download (CyProduct, 2021)
DiscoveryAI SAFIRE	Machine learning drug discovery plaform	Online (DiscoveryAI SAFIRE, 2026)
FP-ADMET	Fingerprint-based machine learning	Free download (FP-ADMET, 2021)
NERDD	Online platform for various opensource ADMET predictions	Free online (NERDD, 2026)
OCHEM	Machine learning (SVMs, RF, NNs)	Free online (OCHEM, 2013)
pkCSM	Graph-based signatures to train predictive models	Free online (pkCSM, 2015)
PredMS	RF machine learning to predict metabolic stability	Free online (PredMS, 2022)
Pred-Skin	Naïve Bayes, RF and SVM machine learning for skin sensitization potential of chemical substances	Free online (PRED, 2026)
ProTox 3.0	Fragment similarity-based CLUSTER cross-validation machine-learning, based on a total of 61 models for the prediction of toxicity endpoints	Free online (ProTox, 2024)
VCCLab (ALOGPS 2.1)	Associative neural networks (ASNNs) to predict the lipophilicity (logP) and aqueous solubility (logS)	Free online (ALOGPS 2.1, 2026)
XenoSite Web	Machine learning for predictions on how small-molecules become toxic after metabolism by liver enzymes	Free online (XenoSite, 2020)

*All models are used for prediction of multiple ADMET end points unless otherwise specified.

The ADMET tools listed in Table 3 demonstrate the rapid expansion of accessible, web-based predictive platforms, spanning graph neural networks (GNNs), classical ML models, DL-based toxicity predictors, and hybrid cheminformatics approaches. Graph-based architectures such as Chemprop-RDKit, DMPNN, CLMGraph, and pkCSM signatures now underpin several widely used platforms (e.g., ADMET-AI, ADMETlab 3.0, admetSAR 3.0), learning molecular features directly from graphs and reducing reliance on hand-crafted descriptors. Other tools, including ADME@NCATS, OCHEM, pkCSM, ProTox 3.0, and XenoSite, extend earlier SVM-, RF-, and NN-based methodologies across multiple endpoints, while specialised resources such as CANDID-CNSTM or CYProduct focus on specific ADMET liabilities.

Most tools in Table 3 are freely accessible and require minimal computational expertise, enabling rapid early-stage triage based on predicted solubility, permeability, metabolic stability, or toxicity. Their scope and methodology, however, vary considerably: platforms like NERDD and OCHEM aggregate multiple models and support user-defined datasets, whereas tools such as Fp-ADMET, Pred-Skin, and PredMS address focused endpoints. Only a subset provides uncertainty estimation or applicability-domain information, which is essential for assessing prediction reliability.

Overall, Table 3 illustrates a shift towards lightweight, cloud-based ADMET modelling that supports routine medicinal chemistry decision-making. These tools are most effective when used within an iterative workflow, applying early *in silico* filters, followed by targeted experimental characterisation, while maintaining awareness of chemical-space limitations and endpoint-specific constraints. Although increasingly influential in early optimisation, these methods should be applied with appropriate caution, particularly as their impact on downstream attrition has yet to be established.

AI is also enhancing PAINS filtering, by moving beyond rigid substructure alerts toward DL-based, context-aware predictions. Tools such as DeepChemProp (2024), Chemprop (2024), ADMETlab 2.0 (2021), MolAICal (2026), and DeepTox (2026) integrate PAINS evaluation into broader ADMET profiling frameworks, improving specificity and reducing false positives (Baell and Nissink, 2018).

Importantly, AI-enabled *in silico* models may eventually help reduce reliance on certain animal studies by improving early prediction of human-relevant properties (Rudroff, 2024), although such reductions remain prospective and have not yet been demonstrated across drug-development pipelines. Combined with microphysiological systems (organ-on-a-chip, 3D tissues), AI may improve prediction of human-relevant ADMET outcomes. Regulatory agencies have begun recognising the potential of AI-based models for early-stage assessment, noting their promise for reducing costs and improving public health outcomes (Rudroff, 2024). The FDA, for example, has recently stated that “by leveraging AI-based computational modelling, human organ model-based lab testing, and real-world human data, we can get safer treatments to patients faster and more reliably, while also reducing R&D costs and drug prices. It is a win-win for public health and ethics”. (Administration F. a. D., 2025).

### 3.7 Drug synthesis

Synthetic route design is a critical yet time-consuming and resource-intensive phase of drug discovery (Blakemore et al., 2018). AI is reshaping retrosynthesis through graph neural networks, transformer-based models, and template-free DL approaches that predict reaction outcomes and propose feasible synthetic routes (Fu and Chen, 2025). In retrosynthesis, GNNs learn reaction rules from atom-mapped examples, while transformer models treat reaction SMILES like a language translation task, from products back to plausible precursors. In self-driving labs (SDLs), machine learning integrates with automation and robotics to optimise reaction conditions and perform closed-loop experimentation (Abolhasani and Kumacheva, 2023). Multi-objective optimisation (e.g., yield, cost, safety, sustainability) can be encoded directly into the optimisation loop, reducing trial-and-error.

Moreover, concerns around purely data-driven retrosynthesis have motivated hybrid AI frameworks that incorporate chemical rules alongside learned representations, improving interpretability and feasibility in complex synthetic planning (Button et al., 2019).

### 3.8 Real world applications of AI in CADD

As AI is influencing pharmaceutical R&D beyond discovery, including synthesis optimisation, supply-chain management, and manufacturing, several AI-facilitated small-molecule programmes have advanced into clinical trials, indicating growing translational impact across indications such as oncology, fibrosis, inflammatory diseases, neurodegeneration, and metabolic disorders (Serrano et al., 2024). Table 4 summarises representative AI-enabled small-molecule candidates currently in clinical trials. The complete dataset is provided in the *Supplementary Information* (Supplementary Table S3). As with Table 1 and Supplementary Table S1, Table 4 and Supplementary Table S2 reflects programmes in which AI/ML tools contributed to early-stage discovery or optimisation; however, these examples should not be interpreted as evidence that AI improves clinical success rates or reduces attrition. In all cases, AI methods supported target prioritisation, scaffold generation, or multi-parameter optimisation, while progression into the clinic relied on extensive medicinal chemistry, translational pharmacology, and experimental validation. The predominance of oncology programmes largely reflects the data-rich nature of kinase targets and the availability of structural and phenotypic datasets, rather than demonstrating that AI is inherently more effective in this therapeutic area. Thus, Table 4 should be interpreted as documenting the types of discovery problems where AI tools have been adopted, rather than as evidence of improved downstream efficiency or clinical performance.

**TABLE 4:** Representative AI-facilitated small-molecule clinical candidates (Pun et al., 2023; Fu and Chen, 2025; Kp Jayatunga et al., 2024).

Drug pipeline	Developer	Target	Indication	Clinical trial stage	ClinicalTrials.gov ID
BMF-219	Biomea Fusion	Menin	Diabetes mellitus type 1/2	Phase II	NCT06152042
BEN-2293	BenevolentAI	Tropomyosin-related kinases	Atopic dermatitis	Phase I/II	NCT04737304
EXS-617 (REC-617)	Exscientia (now Recursion)	CDK7	Advanced solid tumours	Phase I/II	NCT05985655
EXS-4318	Exscientia (Bristol Myers-Squibb)	PKC-theta	Inflammatory diseases	Phase I/II	NCT05760937
INS018-055	InSilico Medicine	TNIK	Idiopathic pulmonary fibrosis	Phase II	NCT05975983
ISM3412	InSilico Medicine	MAT2A	Locally advanced/metastatic solid tumours	Phase I	NCT06414460
NEU-411	Neuron23	LRRK2	Parkinson’s disease	Phase II	NCT06680830
NDI-034858	Nimbus Therapeutics	TYK2	Plaque psoriasis	Phase II	NCT04999839
REC-2282	Recursion	HDAC	Neurofibromatosis type 2	Phase II/III	NCT05130866
RLY-4008	Elevar Therapeutics	FGFR2	Intrahepatic cholangiocarcinoma (ICC) and other advanced solid tumours	Phase I/II	NCT04526106
SGR-1505	Schrödinger	MALT1	Mature B-cell neoplasms	Phase I	NCT05544019
GSBR-1290	Structure Therapeutics	GLP-1R	Obesity	Phase II	NCT06703021

The clinical-stage candidates in Table 4 and Supplementary Table S2 illustrate how AI-enabled design is contributing to early drug-development efforts across oncology, inflammatory diseases, fibrosis, neurodegeneration, and metabolic disorders. Oncology remains the most represented area, driven largely by the wealth of structural and chemogenomic data available for kinases and other well-characterised signalling nodes. Similar patterns are observed across inflammatory, metabolic, and neurological indications, in which AI tools support target prioritisation, scaffold generation, or multiparameter optimisation in data-rich contexts.

Company representation reflects the diversity of AI-enabled strategies currently deployed. Platforms such as those developed by Exscientia, Insilico Medicine, Recursion, Nimbus Therapeutics, Relay Therapeutics, and Schrödinger integrate DL models with structure-based design, phenotypic screening, or automated medicinal-chemistry workflows. Some programmes arise from imaging-based phenomics (e.g., Recursion), others from generative chemistry pipelines (e.g., Insilico and Exscientia), and others from structure-focused ML platforms (e.g., Relay and Schrödinger).

The molecular targets represented, including kinases, epigenetic regulators, protein-protein interaction modulators, GPCRs, and emerging or historically under-addressed target classes, demonstrate that AI is typically deployed where structural, biophysical, or chemogenomic data are sufficiently rich to support reliable modelling. Conversely, only a small subset of programmes focuses on less-characterised protein families, underscoring the ongoing challenge that data scarcity poses for robust AI-driven discovery.

Overall, Table 4 and Supplementary Table S2 indicate that AI is increasingly used to support early design decisions in mechanistically tractable biological spaces, with most candidates residing in Phase I or early Phase II. As noted earlier, these cases document AI involvement in early discovery, not improved downstream clinical performance. While AI can accelerate hypothesis generation and prioritisation, successful translation continues to depend on extensive medicinal chemistry, pharmacology, and carefully designed experimental validation. Indeed, despite these advances, no approved small-molecule drug can yet be unambiguously attributed to a primarily AI-led, end-to-end discovery workflow. Limitations include model sensitivity to data quality (Cavasotto et al., 2024), lack of transparency due to black-box architectures (Hassija et al., 2024), dependence on expert interpretation, and challenges regarding intellectual property and regulatory guidance (Fu and Chen, 2025). Moreover, model generalisability remains limited, necessitating approaches such as transfer learning using pretrained models like ChemBERTa and MolBERT (Altae-Tran et al., 2017; McInnes et al., 2020; Li and Jiang, 2021; Sadaghiyanfam et al., 2025). Transfer learning reuses knowledge from models trained on large, related datasets and fine-tunes them on smaller, task-specific datasets, often boosting performance when labelled data are scarce.

### 3.9 Limitations and challenges of AI-based methods

ML-enabled drug discovery faces several challenges, particularly related to data quality. Sparse datasets hinder reliable 3D-QSAR construction (Vamathevan et al., 2019), while heterogeneous data across assays complicates model integration (Shah et al., 2022). Variability in chemical identifiers highlights the need for harmonisation across databases (Shah et al., 2022), and experimental variability can lead to conflicting activity readouts, further complicating model training (Shah et al., 2022).

Activity cliffs, structurally similar molecules with dramatically different activities, remain difficult for both ML and DL models. A comparative study showed only marginally improved DL performance, with high uncertainty unless large, well-curated datasets were available (van Tilborg et al., 2022). MoleculeACE, Molecule Activity Cliff Estimation, was developed to assess algorithm performance on activity cliffs and may guide resource allocation in medicinal chemistry (van Tilborg et al., 2022). This challenge exemplifies the broader “vicious circle” of model development (Supplementary Figure S2): accurate ML/AI models require large, high-quality datasets, but generating such datasets experimentally is costly and time-consuming, at odds with the goal of reducing synthesis and assay burden. In such scenarios, the ability to evaluate algorithm reliability on limited datasets offers a potential route to more efficient candidate selection and reduced experimental burden.

Model validation should therefore extend beyond internal cross-validation to include external test sets, scaffold- or time-based splits where appropriate, and prospective experimental evaluation to determine whether performance translates to previously unseen chemical space (Sheridan, 2013). In addition, uncertainty estimation plays a critical role in compound prioritisation, particularly when predictions are made for molecules that differ substantially from those represented in the training data (Moosavi et al., 2020). Methods such as ensemble learning (Angelopoulos and Bates, 2023), Bayesian neural networks, Monte Carlo dropout (Son and Seok, 2026), conformal prediction (Angelopoulos and Bates, 2023), and applicability-domain assessment (Carrió et al., 2014) can provide confidence estimates that help identify unreliable predictions and support more informed decision-making. Incorporating these approaches can improve the robustness and practical utility of AI models in drug discovery by enabling better assessment of prediction reliability and reducing the risk of overinterpreting model outputs.

Reproducibility also benefits from simple practices: versioning datasets/splits, fixing random seeds, specifying software environments, and reporting external-set performance alongside cross-validation.

Beyond data quality and benchmarking, several additional factors currently limit the practical adoption of AI-based methods in drug discovery (Pathak et al., 2025). Model interpretability remains a major challenge, particularly for DL approaches, where high predictive performance does not necessarily reveal which molecular features drive activity or selectivity (Pathak et al., 2025). This can make it difficult for medicinal chemists to rationalise predictions or translate them into experimentally actionable design hypotheses. Dataset bias is another important limitation, as models trained on narrow chemical series, overrepresented targets, or assay-specific conditions may learn patterns that do not generalise beyond the original dataset (Arabi, 2021). Consequently, applicability-domain assessment is essential to determine whether a prediction is being made within a chemical space represented by the training data, or whether the model is extrapolating into poorly supported regions. In this context, uncertainty quantification provides an important complement to point predictions by helping identify low-confidence outputs and prioritise compounds with more reliable predicted profiles (Parrondo-Pizarro et al., 2026). Finally, strong retrospective benchmark performance does not always translate into prospective experimental success, highlighting the need for external validation, scaffold- or time-split testing, and prospective synthesis and assay confirmation before AI-derived predictions can be considered practically useful.

The growing diversity of DL-based VS methods has also created challenges in method selection. Tools such as EvaluationMaster aim to benchmark protocols and identify optimal algorithms for specific SBVS tasks (Shen et al., 2025). However, prospective validation remains essential, and no ML- or DL-based ultra-large VS campaign has yet yielded an approved drug.

Together, these limitations highlight that AI should be viewed as a powerful aid, not a replacement, for rigorous experimental design, domain expertise, and transparent model benchmarking in modern drug discovery.

## 4 Ethical and regulatory considerations

As AI and ML methods become increasingly integrated into drug discovery, a number of ethical, legal, and regulatory considerations have emerged. These issues have been discussed in several recent reviews (Arabi, 2021; Kokudeva et al., 2024; Ocana et al., 2025; Anastasopoulou et al., 2024; Niazi, 2023; Sahil et al., 2024), and they span concerns related to employment, intellectual property rights, transparency, accountability, data quality, regulatory alignment, market competition, and environmental impact. Addressing these challenges is essential to ensure that AI-enabled drug discovery progresses responsibly and sustainably. Moreover, data quality issues also impact traditional, non-AI-drive QSAR models.

### 4.1 Employment and workforce transition

The integration of AI into drug discovery raises concerns about potential job displacement, particularly among chemical and biological technicians, due to reduced reliance on manual wet-lab workflows. However, demand for scientists with expertise at the interface of programming, chemistry, and biology is increasing, and workforce transitions may be supported through targeted training initiatives (Anastasopoulou et al., 2024). Regulatory frameworks that enable a gradual and well-managed transition, without hindering innovation, are therefore needed (Anastasopoulou et al., 2024; Niazi, 2023). Regulatory frameworks that enable a gradual and well-managed transition, without hindering innovation, are therefore needed (Anastasopoulou et al., 2024; Niazi, 2023). Regulatory frameworks that enable a gradual and well-managed transition, without hindering innovation, are therefore needed (Anastasopoulou et al., 2024; Niazi, 2023). Regulatory frameworks that enable a gradual and well-managed transition, without hindering innovation, are therefore needed (Anastasopoulou et al., 2024; Niazi, 2023). Regulatory frameworks that enable a gradual and well-managed transition, without hindering innovation, are therefore needed (Niazi, 2023).The integration of AI into drug discovery raises concerns about potential job displacement, particularly among chemical and biological technicians, due to reduced reliance on manual wet-lab workflows. However, demand for scientists with expertise at the interface of programming, chemistry, and biology is increasing, and workforce transitions may be supported through targeted training initiatives (Anastasopoulou et al., 2024). Regulatory frameworks that enable a gradual and well-managed transition, without hindering innovation, are therefore needed (Anastasopoulou et al., 2024; Niazi, 2023). Regulatory frameworks that enable a gradual and well-managed transition, without hindering innovation, are therefore needed (Anastasopoulou et al., 2024; Niazi, 2023). Regulatory frameworks that enable a gradual and well-managed transition, without hindering innovation, are therefore needed (Anastasopoulou et al., 2024; Niazi, 2023). Regulatory frameworks that enable a gradual and well-managed transition, without hindering innovation, are therefore needed (Niazi, 2023).

Automated laboratory systems, such as the Robot Scientist Eve, exemplify this shift. Eve is able to conduct high-throughput screening (∼10,000 compounds/day), build ML-based 3D-QSAR models, and iteratively validate predictions in a closed cycle (Sparkes et al., 2010). Its use in identifying TNP-470 as an inhibitor of *P. vivax* DHFR (IC_50_ = 0.16 µM, with no inhibition of human DHFR) illustrates both the potential and the associated ethical questions regarding employment, intellectual property, and responsibility (Williams et al., 2015).

Beyond drug discovery, AI is reshaping pharmaceutical operations more broadly. For example, Novo Nordisk reported reducing regulatory documentation preparation from 15 weeks to under 10 min using Anthropic’s Claude 3.5 model (OpenTools, 2025). Although unrelated to CADD, this demonstrates the transformative impact of AI across the industry. This trend aligns with the Future of Jobs Report 2023, (World Economic Forum, 2023), which predicts that while AI will lead to job losses in some roles, it will also generate new employment opportunities, resulting in a net positive effect. Importantly, AI and big-data training rank among the highest corporate priorities (World Economic Forum, 2023), which may help to offset job displacement.

### 4.2 Intellectual property rights

Intellectual property rights (IPR) represent a major unresolved issue in AI-enabled drug discovery. The *Thaler v. Vidal* case in the United States ruled that an AI system cannot be listed as an inventor on a patent, and that a natural person must always be identified as the inventor (U.S. Court of Appeals for, 2022). This has raised concerns among pharmaceutical companies about the patent eligibility of therapeutics discovered through AI/ML pipelines, as current legislation does not clarify whether ownership belongs to AI developers, AI owners, or end-users (Sahil et al., 2024).

To mitigate these uncertainties, some companies publish discoveries pre-emptively to prevent competitors from filing patents, while others integrate AI start-ups directly into their R&D divisions (Sahil et al., 2024). A clear, harmonised regulatory framework will be essential, as patent protection remains central to enabling commercialisation and further investment in research.

### 4.3 Transparency, explainability, and accountability

Transparency refers to the ability to understand how an AI model makes decisions, while explainability concerns the extent to which these decisions can be interpreted by humans (Chen, 2023; Niazi, 2025). Both principles are vital for drug discovery, yet difficult to guarantee because many AI models, particularly deep neural networks, operate as “black boxes,” making their internal logic opaque (Chen, 2023; Niazi, 2025).

Responsibility and accountability represent closely related challenges. Although an AI model may guide decisions, its outputs depend entirely on how it has been designed and trained; therefore, responsibility lies with the human developers, owners, and/or users (Niazi, 2023; Chen, 2023).

If a drug approved on the basis of AI-guided evidence later exhibits severe side effects, liability may rest with users who bypassed appropriate testing, or with the model developers, an issue complicated by the absence of specific laws governing AI in drug development (Sahil et al., 2024).

### 4.4 Bias, data quality, and fairness

Bias may arise when small or low-quality datasets, or ones not adequately representative of biological and demographic diversity, are used to train AI/ML models or build QSAR models (Sahil et al., 2024). If the data are limited in quantity or derive from heterogeneous sources employing different formats and data collection protocols, their quality may be compromised (Gangwal and Lavecchia, 2025). The datasets may be small, insufficiently diverse (e.g., data derived from a single type of biological assay), outdated, or heavily affected by experimental variability (particularly when complex biological assays are involved and the results varies depending on the environment and the operators) (Li et al., 2025). In the case of QSAR modelling, these limitations may result in a reduced ability to accurately predict the activity of outliers (structural outliers and activity cliffs) (Li et al., 2025) and, more generally, in both QSAR modelling and AI-based large-scale virtual screening poor-quality data can lead to the prioritisation and synthesis of compounds that ultimately fail during clinical development, resulting in significant losses of time and financial resources for both academia and industry. Another major concern is algorithmic bias, which arises when training datasets do not adequately represent the diversity of the target population (Gangwal and Lavecchia, 2025). For example, if historically underrepresented groups, such as women or ethnic minorities, are insufficiently represented in the training data, drug candidates identified through these models may exhibit non optimal efficacy or safety profiles in these populations (Sahil et al., 2024). In the context of rare diseases, the challenge is even more pronounced, as data quality is inherently constrained by the scarcity of available patient data (Gangwal and Lavecchia, 2025). Ensuring diverse, high-quality datasets is therefore essential to minimise algorithmic bias and promote equitable healthcare outcomes.

### 4.5 Regulatory guidelines and global alignment

Regulatory frameworks for AI in drug discovery remain fragmented, with the potential to hinder adoption and slow international collaboration (Mirakhori and Niazi, 2025). The U.S. FDA’s discussion paper on AI/ML in drug development addresses issues such as data bias, transparency, and the black-box nature of AI, recommending the use of explainable models and robust human oversight (Mirakhori and Niazi, 2025). These guidelines also discuss AI-driven clinical-trial design, including decentralised clinical trials, and emphasise standards for informed consent, data quality, and bias mitigation (Mirakhori and Niazi, 2025).

The European Medicines Agency (EMA) adopts a more cautious stance, with guidelines that focus on data protection and require compliance with the GDPR, the Data Act, and the AI Act (Mirakhori and Niazi, 2025). Both agencies align with WHO principles stating that AI should augment, not replace, human decision-making and must integrate good manufacturing practices from preclinical to production stages (Mirakhori and Niazi, 2025).

### 4.6 Environmental sustainability

AI-enabled drug-discovery workflows, particularly DL models trained on large datasets, can carry significant computational and environmental costs (Ligozat et al., 2022). Large data transfers, repeated docking or screening cycles, and hyperparameter optimisation all contribute to energy usage, and the training of single large models has been associated with substantial CO_2_ emissions (van Wynsberghe, 2021). In particular, the training of a single deep learning NLP model on GPU-based infrastructure has been estimated to result in approximately 600,000 l b of CO_2_ emissions (van Wynsberghe, 2021). Tools such as Green Algorithms (c) and ML CO_2_ Impact (Machine Learning Emissions Calculator, 2026), allow researchers to estimate the footprint of specific workflows, helping medicinal chemists judge whether model size or screening scale is proportionate to the scientific goal (Ligozat et al., 2022). AI-enabled drug-discovery workflows, particularly DL models trained on large datasets, can carry significant computational and environmental costs (Ligozat et al., 2022). Large data transfers, repeated docking or screening cycles, and hyperparameter optimisation all contribute to energy usage, and the training of single large models has been associated with substantial CO_2_ emissions (van Wynsberghe, 2021). Tools such as Green Algorithms (Green Algorithms, 2026) and ML CO_2_ Impact (Machine Learning Emissions Calculator, 2026), allow researchers to estimate the footprint of specific workflows, helping medicinal chemists judge whether model size or screening scale is proportionate to the scientific goal. (Ligozat et al., 2022).

Sustainability also intersects directly with methodological choices in CADD. Task-specific or pretrained models often deliver suitable performance at a fraction of the cost of foundation-scale architectures, while transfer learning, model distillation, and parameter-efficient fine-tuning further reduce training demands. Cloud-based virtual screening (Table 2) and open-source ADMET tools (Table 3) can additionally leverage shared, optimised hardware, lowering per-user energy overheads compared to local high-performance clusters.

These considerations fall within the broader concept of “Sustainable AI”, encompassing both efforts to reduce the environmental burden of AI systems and the use of AI to advance sustainability objectives (van Wynsberghe, 2021). For CADD practitioners, adopting efficient models, monitoring energy usage, and selecting computational approaches that match the needs of a given project represent practical steps toward integrating AI responsibly while minimising environmental impact.

## 5 Conclusion

Computer-aided drug discovery (CADD) has evolved from early QSAR models into a broad suite of structure-based, ligand-based, and data-driven approaches that now support modern drug-discovery pipelines. These methods have contributed to the discovery and optimisation of multiple approved therapeutics and have improved the efficiency of hit identification, lead optimisation, and ADMET assessment. Advances in AI and ML have further expanded predictive capabilities and accessible chemical space, with tools such as AlphaFold2 and AlphaFold3 reshaping structure-based design and enabling more integrated computational workflows involving SBDD, LBDD, generative modelling, molecular dynamics, and cloud-based virtual screening.

By assembling major open-access platforms for virtual screening and freely available ADMET-prediction tools, this review offers a practical resource to support the immediate adoption of CADD and AI methodologies in medicinal chemistry.

Despite these developments, challenges remain. Although several therapeutics have involved computational or AI-supported methods during discovery, optimisation, or development, the extent of AI contribution varies widely and the definition of an “AI-discovered” or “AI-enabled” therapeutic remains debated. To date, no approved small-molecule therapeutic can yet be definitely attributed to a primarily AI-led, end-to-end discovery workflow, although several AI-facilitated candidates have progressed into clinical evaluation. Historical and contemporary examples demonstrate that successful programmes typically rely on context-dependent combinations of computational modelling, medicinal chemistry and experimental validation rather than single-technique strategies. Limitations related to data quality, dataset bias, restricted applicability domains, poor uncertainty quantification, model opacity, and limited prospective validation continue to affect interpretability, reproducibility, regulatory confidence, and practical adoption. Progress will require high-quality and representative training datasets, better model explainability, and robust validation frameworks, alongside attention to ethical and regulatory considerations such as intellectual-property ownership, accountability, data stewardship, and environmental sustainability.

Looking ahead, interdisciplinary collaboration and continued methodological refinement will strengthen the integration of CADD into routine medicinal-chemistry practice. As computational tools become more transparent, reliable, and efficient, and as regulatory guidance matures, the potential for reduced reliance on animal models may grow, though such impacts remain prospective. With careful experimental validation and responsible deployment, CADD and AI-enabled approaches are well positioned to support the discovery of safer, more effective, and more ethical therapeutic solutions.

Across these developments, three main principles can guide the responsible adoption of AI in CADD and directly address the limitations discussed above. First, as retrospective benchmark performance does not necessarily translate into prospective success, AI-generated hypotheses should be prioritised and triaged using rapid, low-cost experimental assays before committing significant synthetic or biological resources. Second, as dataset bias and applicability-domain constraints can lead to unreliable extrapolation, medicinal chemists should favour models with clearly defined applicability domains, documented uncertainty estimates, and, where possible, mechanistic interpretability. Third, as complex models can increase opacity, computational cost, and validation burden, practitioners should choose the simplest model that meets the scientific need, using lightweight or pre-trained models for routine property prediction and reserving more complex architectures for problems where they provide demonstrated added value. Together, these principles offer a practical pathway for integrating AI into medicinal-chemistry workflows in a way that strengthens decision-making while maintaining scientific rigour and accountability.

## Footnotes

1 Green tick accessed from https://favpng.com Protein structural model produced internally using MOE accessed at https://www.chemcomp.com/en/Products.htm. Rat Clip Art accessed at https://clipground.com/rat-clipart-transparent.html Human biological outline, pipette and conical flask owned by ChemDraw https://revvitysignals.com/products/research/chemdraw.
