# Supplementary material for: Computer-aided drug discovery: historical foundations, practical AI tools, and emerging ethical considerations
Source: Front Pharmacol. 2026 Jun 24;17:1866562. doi: 10.3389/fphar.2026.1866562 (PMC13341473; doi:10.3389/fphar.2026.1866562)
Supplement: Supplementary file 1 [file DataSheet1.pdf]

Supporting Information

Computer-aided drug discovery: historical foundations, practical AI tools, and emerging ethical considerations

Rebekah Clarke [1,2], Giuseppe Palazzo [1], Jiri Ruzicka [2], Salvatore Ferla [1], and Marcella Bassetto [2]*

[1] Medical School, Faculty of Medicine, Health and Life Science, Swansea University, Swansea, UK SA2 8PP, UK

[2] School of Pharmacy and Pharmaceutical Sciences, Cardiff University, Cardiff CF10 3NB, UK

* Correspondence: M.B., bassettom1@cardiff.ac.uk

Contents

Figure S1 *Page 2*

Figure S2 *Page 3*

Table S1 *Pages 4-6*

Table S2 *Pages 7-9*

References *Page 10*

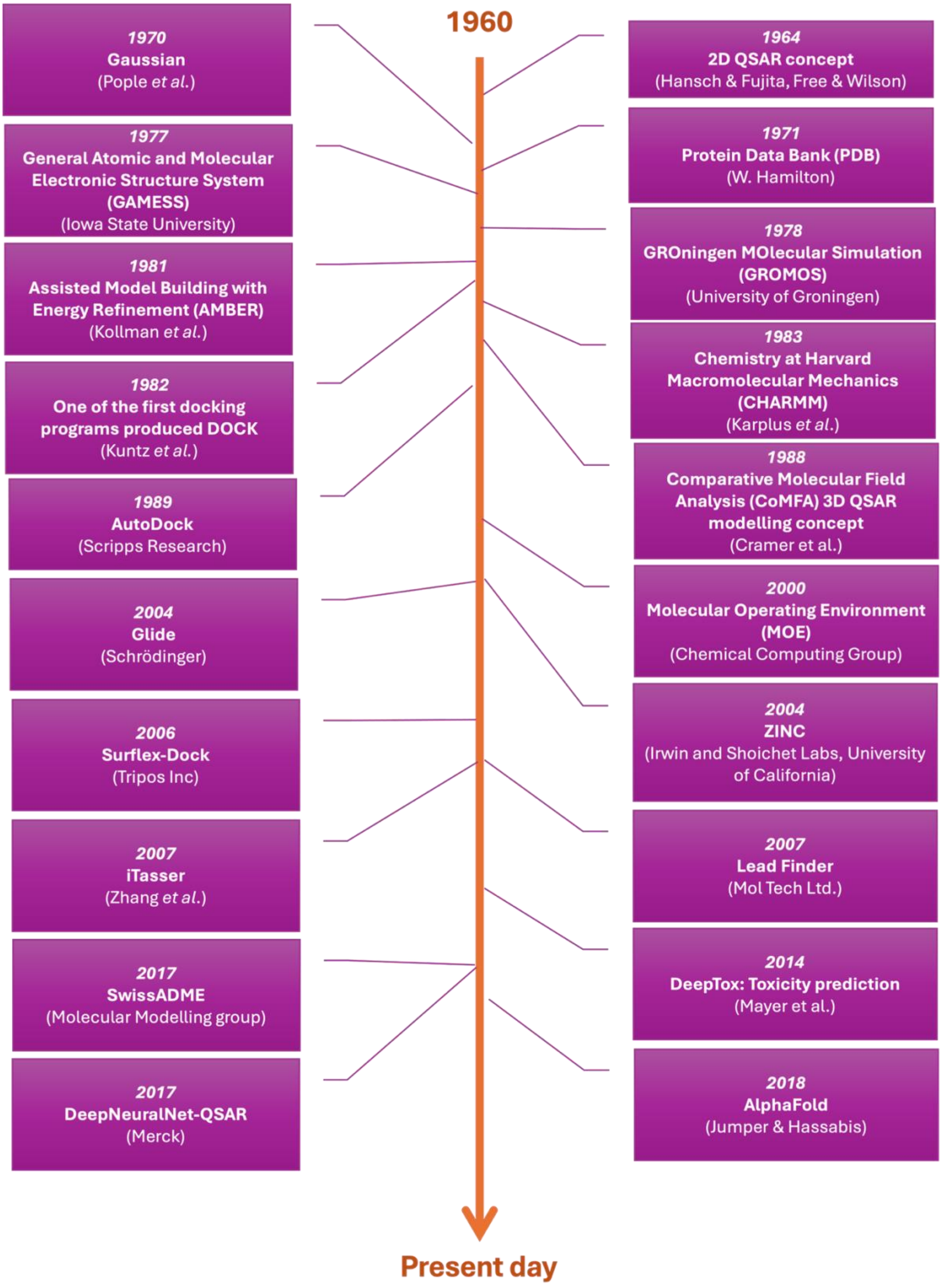

Figure S1. A timeline of key milestones in the progress of technologies and concepts involved in CADD.

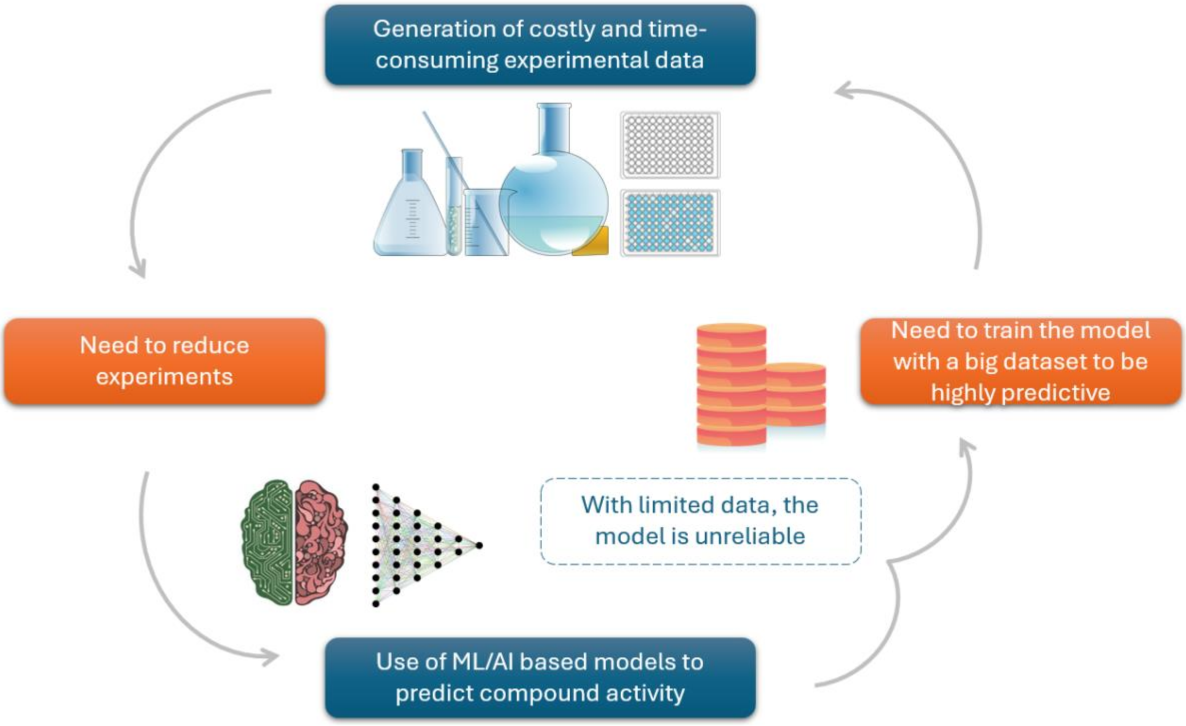

Figure S2. "The vicious circle" of data generation and ML/AI based models in compound activity prediction.

Drug	Therapeutic Application	CADD Approach	FDA Approval Date
**Captopril**	Angiotensin-converting enzyme (ACE) inhibitor (hypertension)	QSAR	1981
**Norfloxacin**	Bacterial DNA gyrase inhibitor (antibacterial)	QSAR	1983
**Flurbiprofen**	Nonsteroidal anti-inflammatory drug (NSAIDs)	Molecular docking	1987
**Epalrestat**	Non-competitive and reversible aldose reductase inhibitor (diabetic neuropathy)	Molecular docking and structure-based VS	1992
**Cladribine**	Nucleoside deoxyadenosine mimic (lymphocytic leukaemia's and multiple sclerosis)	Structure-based VS with docking studies	1993
**Dorzolamide**	Carbonic anhydrase inhibitor (glaucoma and ocular hypertension)	Fragment-based screening	1994
**Losartan**	Angiotensin II receptor antagonist (hypertension)	LBDD, pharmacophore modelling and SAR optimisations	1995
**Saquinavir**	Inhibitor of HIV I protease (HIV)	SBDD	1995
**Indinavir**	Inhibitor of HIV I protease (HIV)	SBDD with molecular dynamics and X-ray crystallography	1996
**Ritonavir**	Inhibitor of HIV I protease (HIV)	SBDD, LBDD, SAR optimisations	1996
**Donepezil**	Cholinesterase inhibitor (Alzheimer's)	3D-QSAR and docking studies	1996
**Nelfinavir**	Inhibitor of HIV I protease (HIV)	SBDD	1997
**Zolmitriptan**	Selective serotonin 5-HT1B and 5-HT1D receptor agonist (Migraines)	Pharmacophore modelling and LBDD	1997
**Delavirdine**	HIV reverse transcriptase (HIV)	SBDD, LBDD, SAR optimisation	1997
**Efavirenz**	Non-nucleoside reverse transcriptase (HIV)	SBDD	1998
**Tirofiban**	Glycoprotein IIb/IIIa inhibitors (antiplatelet drug)	LBDD with pharmacophore screening	1999
**Oseltamivir**	Antiviral (influenza A and B)	SBDD	1999
**Zanamivir**	Antiviral (influenza A and B)	SBDD	1999
**Amprenavir**	Inhibitor of HIV protease (HIV)	Molecular modelling and MD	1999
**Lopinavir**	Inhibitor of HIV protease (HIV)	SBDD	2000
**Eprosartan**	Angiotensin II receptor antagonist (hypertension)	SBDD and LBDD with pharmacophore modelling	2001
**Imatinib**	Tyrosine kinase inhibitor (cancers)	SBDD	2001
**Valsartan**	Angiotensin II receptor blocker (hypertension)	Energy-minimisations and QSAR	2002
**Atazanavir**	Inhibitor of HIV protease (HIV)	Computational mapping of protein binding sites and ligand docking	2003
**Enfuvirtide**	Inhibitor of HIV protease (HIV)	Homology modelling	2003
**Fosamprenavir**	Inhibitor of HIV protease (HIV)	SBDD	2003
**Gefitinib**	EGFR tyrosine kinase inhibitor (certain breast, lung and other cancers)	Structure-based VS	2003
**Erlotinib**	EGFR tyrosine kinase inhibitor (non-small cell lung cancer (NSCLC) and pancreatic cancer)	Structure-based VS	2005
**Tipranavir**	Nonpeptidic HIV protease inhibitor	SBDD with x-ray crystallography and docking	2005
**Darunavir**	Nonpeptidic HIV protease inhibitor	SBDD and LBDD	2006

**Sunitinib**	Tyrosine receptor kinases inhibitor (renal cell carcinoma (RCC) and gastrointestinal stromal tumour (GIST))	Homology modelling and SAR optimisations	2006
**Aliskiren**	Renin inhibitor (primary hypertension)	SBDD with docking simulations	2007
**Ambrisentan**	Type A endothelin receptor (ETA) antagonist (hypertension)	Structure-based VS	2007
**Maraviroc**	CCR5 chemokine receptor antagonist (CCR5-tropic HIV)	Structure-based VS, QSAR and MD simulations	2007
**Nilotinib**	Bcr-Abl Tyrosine Kinase Inhibitor (chronic myeloid leukaemia (CML))	Rational drug design with structural based modelling	2007
**Raltegravir**	HIV integrase inhibitor (HIV)	SBDD with MD simulations	2007
**Boceprevir**	Protease inhibitor (hepatitis C)	SBDD with x-ray crystal structures and transition-state mimetics	2011
**Crizotinib**	ALK-receptor tyrosine kinase inhibitor (NSCLC)	SBDD with co-crystal structures	2011
**Rilpivirine**	Non-nucleoside reverse transcriptase inhibitor (HIV)	SBDD with mechanistic studies and transition state isostere modelling	2011
**Rivaroxaban**	Factor Xa inhibitor (anticoagulant)	SBDD with HTS and SAR	2011
**Telaprevir**	NS3/4A serine protease inhibitor (hepatitis C)	LBDD with structure-based optimisations	2011
**Dolutegravir**	HIV integrase inhibitor (HIV)	PBDD	2013
**Grazoprevir**	NS3/4A serine protease inhibitor (hepatitis C)	SBDD with docking simulations	2016
**Lifitegrast**	LFA-1/ICAM-1 interaction inhibitor (dry eye disease)	SBDD	2016
**Rucaparib**	PARP-1 inhibitor (anti-cancer)	LBDD	2016
**Venetoclax**	BCL-2 inhibitor (leukaemia treatment)	FBDD	2016
**Acalabrutinib**	Bruton tyrosine kinase inhibitor (chronic lymphocytic leukaemia (CLL))	SBDD with docking	2017
**Betrixaban**	Factor Xa inhibitor (anticoagulant**)**	Molecular docking	2017
**Brigatinib**	Anaplastic lymphoma kinase (ALK) and epidermal growth factor receptor (EGFR) inhibitor (NSCLC)	Homology modelling and molecular docking studies	2017
**Copanlisib**	PI3K inhibitor (follicular lymphoma)	SBDD including X-ray crystallography and docking. LBDD based on lead scaffold	2017
**Vaborbactam**	β-lactamase inhibitor (gram-negative bacterial infections)	Molecular docking studies with MD	2017
**Abemaciclib**	CDK inhibitor selective for CDK4 and CDK6 (breast cancer)	SBDD with SAR studies	2017
**Apalutamide**	Androgen receptor inhibitor (prostate cancer)	SBDD with SAR studies	2018
**Dacomitinib**	Selective and irreversible inhibitor of EGFR (NSCLC)	SBDD with SAR optimisation	2018

**Duvelisib**	PI3K inhibitor (anti-cancer)	SBDD with molecular docking and VS. LBDD with SAR for lead optimisation	2018
**Ivosidenib**	IDH1 inhibitor (AML, cholangiocarcinoma)	LBDD with SAR studies	2018
**Larotrectinib**	Inhibitor of tropomyosin kinase receptors (cancers: tissue agnostic)	LBDD with SAR and crystal-binding mode similarity studies	2018
**Lorlatinib**	ALK and ROS1 inhibitor (NSCLC)	SBDD with co-crystal structure modelling and pharmacokinetic property analysis	2018
**Talazoparib**	PARP inhibitor (advanced breast cancer with germline BRCA mutations)	SBDD with cocrystal structure modelling and SAR for lead optimisation	2018
**Darolutamide**	Selective competitive silent antagonist of the androgen receptor (AR) (non-metastatic castration-resistant prostate cancer)	SBDD with docking and molecular dynamics	2019
**Entrectinib**	Tyrosine kinase inhibitor of the TRK A, B and C, ROS1 and ALK (ROS1-positive NSCLC and NTRK fusion-positive solid tumours)	SBDD and SAR optimisations	2019
**Erdafitinib**	FGFR inhibitor (metastatic or locally advanced bladder cancer with an FGFR3 or FGFR2 alteration)	FBDD and SBDD	2019
**Fedratinib**	Semi-selective inhibitor of Janus kinase 2 (JAK-2) (myelofibrosis)	SBDD with VS and molecular docking	2019
**Selinexor**	Selective inhibitor of nuclear export (multiple myeloma)	SBDD with consensus induced fit docking	2019
**Zanubrutinib**	Bruton's tyrosine kinase inhibitor (B-cell cancers)	FBDD and SBDD	2019
**Alpelisib**	Alpha-specific PI3K inhibitor (breast cancer)	Molecular docking studies	2019
**Siponimod**	Selective sphingosine-1-phosphate receptor modulator (multiple sclerosis)	SBDD with machine learning	2019
**Sotorasib**	RAS GTPase inhibitor for (non-small cell lung cancer)	FBDD	2021
**Abrocitinib**	Janus kinase inhibitor (eczema)	SBDD with QSAR	2022
**Tepotinib**	c-Met (NSCLC)	SBDD with docking studies	2022
**Inavolisib**	PI3K inhibitor (anti-cancer)	SBDD with docking studies	2024

Table S1. A comprehensive table of clinically approved drugs which have been discovered through CADD approaches; HTS – High Throughput Screening; MD – Molecular Dynamics; LBDD – Ligand-Based Drug Design; PBDD – Pharmacophore-Based Drug Design; SBDD – Structure-Based Drug Design; VS – Virtual Screening; SAR – Structural Activity Relationship; QSAR – Quantitative Structural Activity Relationship.

Drug pipeline	Developer	Target	Indication	Clinical trial stage	ClinicalTrials.gov ID
**AO-252**	A2A Pharmaceuticals	TACC3	Ovarian cancer, triple-negative breast cancer (TNBC)	Phase I	NCT06136884
**BMF-219**	Biomea Fusion	Menin	Diabetes mellitus type 1/2	Phase II	NCT06152042
**AC-676**	Accutar Biotechnology	BTK	B-cell malignancies	Phase I	NCT05780034
**AC-699**	Accutar Biotechnology	Oestrogen receptor	Breast cancer	Phase I	NCT05654532
**ATH-63**	Athos Therapeutics	G9A	Ulcerative colitis and Crohn's disease	Phase I	NCT05807971
**AU-409**	Auransa	RNA Transcription modulation (exact details undisclosed)	Advanced hepatocellular carcinoma	Phase I	NCT05791448
**BEN-2293**	BenevolentAI	Tropomyosin-related kinases	Atopic dermatitis	Phase I/II	NCT04737304
**BEN-8744**	BenevolentAI	PDE10	Inflammatory bowel diseases	Phase I	NCT06118385
**BEN-2001**	BenevolentAI	Histamine H3 receptor	Excessive daytime sleepiness (EDS) in Parkinson's disease (PD)	Phase II	NCT03194217
**Talabostat (BXCL-701)**	BioXcel Therapeutics	Dipeptidyl peptidases (DPP)	Acute myeloid leukaemia	Phase I	NCT05703542
**BDTX-1535**	Black Diamond Therapeutics	EGFR	Glioblastoma or non-small cell lung cancer	Phase I/II	NCT05256290
**BDTX-4933**	Black Diamond Therapeutics	BRAF Class I/ II/III	Recurrent advanced/metastatic non-small cell lung cancer (NSCLC)	Phase I	NCT05786924
**INDV-2000**	Indivior Inc.	Orexin-1 receptor	Opioid-use disorder	Phase I	NCT04976855
**EXS-617 (REC-617)**	Exscientia (now Recursion)	CDK7	Advanced solid tumours	Phase I/II	NCT05985655
**EXS-4318**	Exscientia (Bristol Myers-Squibb)	PKC-theta	Inflammatory diseases	Phase I/II	NCT05760937
**EXS-74539**	Exscientia	LSD1	Acute myeloid leukaemia and small-cell lung cancer	Phase I/II	Awaiting submission
**EXS-73565**	Exscientia	MALT1	Haematological cancers	Phase I	Awaiting submission
**HST-1011**	HotSpot Therapeutics	CBL-B	Advanced solid tumours	Phase I/II	NCT05662397
**INS018-055**	InSilico Medicine	TNIK	Idiopathic pulmonary fibrosis	Phase II	NCT05975983
**ISM3412**	InSilico Medicine	MAT2A	Locally advanced/metastatic solid tumours	Phase I	NCT06414460
**ISM8207**	InSilico Medicine	QPCTL	Relapsed/refractory B-cell lymphoma	Phase I	NCT06445517
**ISM6331**	InSilico Medicine	TEAD	Advanced/metastatic malignant mesothelioma	Phase I	NCT06566079

**ISM5411**	InSilico Medicine	PHD1 and PHD2	Irritable bowel syndrome	Phase I	NCT06012578
**ISM5939**	InSilico Medicine	ENPP1	Advanced and/or metastatic solid tumours	Phase I	NCT06724042
**NX-13**	Landos Biopharma	NLRX1 pathway	Ulcerative colitis	Phase I	NCT04862741
**NIM-1324**	NImmune Biopharma	LANCL2	Systemic lupus erythematosus (SLE)	Phase I	NCT05019950
**MDR-001**	MindRank AI	GLP-1R	Obesity	Phase I/II	NCT06778850
**NEU-411**	Neuron23	LRRK2	Parkinson's disease	Phase II	NCT06680830
**NDI-010976**	Nimbus Therapeutics	ACC	Fractional *de novo* lipogenesis (DNL)	Phase I	NCT02876796
**NDI-034858**	Nimbus Therapeutics	TYK2	Plaque psoriasis	Phase II	NCT04999839
**NDI-101150**	Nimbus Therapeutics	HPK1	Solid tumours	Phase I/II	NCT05128487
**NDI-219216**	Nimbus Therapeutics	WRN	Advanced solid tumours	Phase I/II	NCT06898450
**NB-001**	Nobias Therapeutics	AC1	22q11 deletion syndrome	Phase II	NCT05290493
**PHI 101**	Pharos iBio Co.	FLT3	Relapsed or refractory acute myeloid leukaemia	Phase I	NCT04842370
**REC-4881**	Recursion	MEK1/2	Familial adenomatous polyposis	Phase I	NCT05552755
**REC-1245**	Recursion	RBM39	Solid tumours and lymphoma	Phase I/II	NCT06678659
**REC-2282**	Recursion	HDAC	Neurofibromatosis type 2	Phase II/III	NCT05130866
**REC-994**	Recursion	CCM2	Cerebral cavernous malformation	Phase II	NCT05085561
**REC-3964**	Recursion	Toxin B	*Clostridioides difficile* Infection	Phase II	NCT06536465
**RLY-1971**	Relay Therapeutics (now Genentech own rights)	SHP2	Metastatic solid tumours	Phase I	NCT04252339
**RLY-2608**	Relay Therapeutics	PI3Kα	PIK3CA related overgrowth spectrum (PROS) and malformations	Phase II	NCT06789913
**RLY-4008**	Elevar Therapeutics	FGFR2	Intrahepatic cholangiocarcinoma (ICC) and other advanced solid tumours	Phase I/II	NCT04526106
**RLY-5836**	Relay Therapeutics	PIK3CA mutated	Advanced breast cancer and other solid tumours	Phase I	NCT05759949
**SGR-2921**	Schrödinger	CDC7	Relapsed/refractory acute myeloid leukaemia or myelodysplastic syndrome	Phase I	NCT05961839
**SGR-1505**	Schrödinger	MALT1	Mature B-cell neoplasms	Phase I	NCT05544019
**SOM0226**	SOM Innovation Biotech	TTR	Familial amyloid polyneuropathy	Phase II	NCT02191826
**SOM-3355**	SOM Innovation Biotech	VMAT2	Huntington's disease	Phase II	NCT05475483
**GSBR-1290**	Structure Therapeutics	GLP-1R	Obesity	Phase II	NCT06703021
**TOS-358**	Totus Medicines	PI3K-alpha	Select solid tumours	Phase I	NCT05683418
**VRG-50635**	Verge Genomics	PIKfyve	Sporadic and familial amyotrophic lateral sclerosis	Phase I	NCT06215755
**OPL-0401**	Valo Health	ROCK1/2	Diabetic retinopathy	Phase II	NCT05393284

Table S2. Active small molecule pipelines in various stages of clinical trials whose discovery was facilitated by AI[1-3]

References

(1) Fu, C.; Chen, Q. The future of pharmaceuticals: Artificial intelligence in drug discovery and development. *Journal of Pharmaceutical Analysis* **2025**, 101248. DOI: 10.1016/j.jpha.2025.101248.
(2) Pun, F. W.; Ozerov, I. V.; Zhavoronkov, A. AI-powered therapeutic target discovery. *Trends in Pharmacological Sciences* **2023**, *44* (9), 561-572. DOI: 10.1016/j.tips.2023.06.010.
(3) Kp Jayatunga, M.; Ayers, M.; Bruens, L.; Jayanth, D.; Meier, C. How successful are AI-discovered drugs in clinical trials? A first analysis and emerging lessons. *Drug Discovery Today* **2024**, *29* (6), 104009. DOI: 10.1016/j.drudis.2024.104009.
